# Analysis of the genotype–phenotype correlation of *MYO15A* variants in Chinese non-syndromic hearing loss patients

**DOI:** 10.1186/s12920-022-01201-3

**Published:** 2022-03-26

**Authors:** Ying Fu, Shasha Huang, Xue Gao, Mingyu Han, Guojian Wang, Dongyang Kang, Yongyi Yuan, Pu Dai

**Affiliations:** 1 Department of Otolaryngology, Head and Neck Surgery, Nanfang Hospital, Southern Medical University, Guangzhou, Guangdong People’s Republic of China; 2 College of Otolaryngology Head and Neck Surgery, National Clinical Research Center for Otolaryngologic Diseases, State Key Lab of Hearing Science, Ministry of Education, Beijing Key Lab of Hearing Impairment Prevention and Treatment, Chinese PLA General Hospital, Chinese PLA Medical School, Beijing, People’s Republic of China; 3 Department of Otorhinolaryngology, Qilu Hospital (Qingdao), Cheeloo College of Medicine, Shandong University, Qingdao, Shandong People’s Republic of China; 4 Department of Otolaryngology, PLA Rocket Force Characteristic Medical Center, Beijing, People’s Republic of China

**Keywords:** *MYO15A*, DFNB3, Hearing loss (HL), Non-syndromic sensorineural hearing loss (NSHL)

## Abstract

**Background:**

Mutations in the *MYO15A* gene are a widely recognized cause of autosomal recessive non-syndromic sensorineural hearing loss (NSHL) globally. Here, we examined the role and the genotype–phenotype correlation of *MYO15A* variants in a cohort of Chinese NSHL cases.

**Methods:**

Eighty-one cases with evidenced *MYO15A* variants from the 2263 Chinese NSHL cases, who underwent next-generation sequencing (NGS), were enrolled in the study. We investigated the association of *MYO15A* variants with the severity, progression and age of onset of hearing loss, as well as compared it to the previous reports in different nationalities. The cases were divided into groups according to the number of truncating variants: 2 truncating, 1 truncating and 1 non-truncating, 2 non-truncating variants, and compared the severity of HL among the groups.

**Results:**

*MYO15A* accounted for 3.58% (81/2263) of all NSHL cases. We analyzed 81 *MYO15A*-related NSHL cases, 73 of whom were with congenital bilateral, symmetric or severe-to-profound hearing loss (HL), however, 2 of them had a postlingual, asymmetric, mild or moderate HL. There were 102 variants identified in all *MYO15A* structural domains, 76.47% (78/102) of whom were novel. The most common types of detected variants were missense (44/102, 43.14%), followed by frameshift (27/102, 26.47%), nonsense (14/102, 13.72%), splice site (10/102, 9.80%), in frame (4/102, 3.92%), non-coding (2/102, 1.96%) and synonymous (1/102, 0.98%). The most recurrent variant c.10245_10247delCTC was detected in 12 cases. We observed that the *MYO15A* variants, located in its N-terminal, motor and FERM domains, led to partial deafness with better residual hearing at low frequencies. There were 34 cases with biallelic truncating variants, 37 cases with monoallelic truncating variants, and 13 cases with biallelic non-truncating variants. The biallelic non-truncating variants group had the least number of cases (12/81), and most of them (10/12) were with profound NSHL.

**Conclusions:**

*MYO15A* is a major gene responsible for NSHL in China. Cases with *MYO15A* variants mostly showed early-onset, symmetric, severe-to-profound hearing loss. This study is by far the largest focused on the evaluation of the genotype–phenotype correlations among the variants in the *MYO15A* gene and its implication in the outcome of NSHL. The biallelic non-truncating *MYO15A* variants commonly caused profound HL, and the cases with one or two truncating *MYO15A* variants tended to increase the risk of HL. Nevertheless, further investigations are needed to clarify the causes for the variable severities and progression rates of hearing loss and the detected *MYO15A* variants in these cases.

**Supplementary Information:**

The online version contains supplementary material available at 10.1186/s12920-022-01201-3.

## Background

Hearing loss (HL) is one of the most common human pathologies that significantly affects the quality of life [1]. About 60% of congenital HL is caused by genetic factors [2, 3]. Non-syndromic sensorineural hearing loss (NSHL) is considered a major cause of HL. To date, mutations in 124 genes have been identified in individuals affected with NSHL, among which mutations in 78 genes were related to autosomal recessive non-syndromic sensorineural hearing loss (ARNSHL), mutations in 51 autosomal dominant genes and 5 X-linked genes were correlated with NSHL (Hereditary Hearing Loss Homepage, http://hereditaryhearingloss.org, updated on 30 August 2021). The most common variations that were found in ARNSHL were in the genes *GJB2, SLC26A4, CDH23, MYO15A* and *OTOF* [4, 5]. Genetic variations in *MYO15A* were considered the third most common cause of ARNSHL in Iran due to prevalent consanguineous marriages [5, 6]. Whereas in the cohort of Korean ARNSHL patients, *MYO15A* mutations were recognized as the fourth most important deafness gene variants after those detected in other genes like *GJB2*, *SLC26A4* and *CDH23* [7, 8].

*MYO15A* (OMIM #602666) is a 71 kb long gene that contains 66 exons. It is localized on chromosome 17p11.2 (chr17:18012020–18083116; hg19 assembly) and encodes the myosin-XV protein with 35,390 amino acids [9]. Myosin proteins are a large family of actin-based molecular motors that bind actin filaments to produce force and motion, thus contributing to the hydrolysis of ATP.

The MYO15A protein contains an N-terminal domain (amino acids (AA) 1–1223), a motor domain (AA 1224–1899), three light-chain binding IQ motifs (AA1909–1942), two myosin-tail homologies 4 domains (MyTH4, AA 2066–2174 and 3051–3161), two band F, ezrin, radixin, myosin domains (FERM, AA 2687–2867 and 3217–3497), an Src-homology-3 domain (SH3, AA 2865– 2959) and a C-terminal PDZ ligand motif [6, 10, 11].

It is reported that *MYO15A* mutations cause sensorineural HL in human autosomal recessive deafness 3 (DFNB3, OMIM #600316) [7]. The DFNB3 locus was discovered in patients from a remote village in Indonesia, where 2.2% (47/2185) of the population was affected by hearing loss [12, 13]. So far, more than 200 *MYO15A* variants have been reported in more than 20 countries and regions, such as Algeria, Arab, Brazil, China, France, Germany, India, Iran, Israel, Japan, Mexico, the Netherlands, Oman, Pakistan, Palestine, Qatar, South Korea, Spain, Tunisia, Turkey and the United States. However, due to the large size of the gene and its many exons, simple techniques for detecting variants are discordant with it. Therefore, the clinical characteristics of *MYO15A* related to NSHL hearing level, age of onset, the degree of progression, associated symptoms and hotspot mutations were not clearly identified. So far, *MYO15A* had been reported sporadically in China. In this study, 81 cases from 74 families identified with at least one *MYO15A* pathogenic or likely pathogenic variants, or uncertain significant variants, diagnosed by next-generation sequencing (NGS) from 2263 Chinese cases with NSHL, were enrolled to analyze the correlation between the *MYO15A* genomic variants and NSHL pathological phenotype. Co-segregation of variants was confirmed in probands and healthy parents, as well as more family members if available, via NGS and Sanger sequencing. This study is by far the largest focused on *MYO15A* variants and their implication in the outcome of NSHL. As well as we were able to detect the gene frequency and the recurrent variant of the *MYO15A* in Chinese patients with NSHL. The association of *MYO15A* variants with hereditary deafness patients, their severity, progression and age of onset was further conducted.

## Methods

### Purpose of test

The performed test aimed to examine the role and the genotype–phenotype correlation of *MYO15A* variants in a cohort of Chinese NSHL patients.

### Subjects and clinical evaluation

There were 2263 participants from 1842 families with NSHL from the Genetic Testing Center for Deafness at the College of Otolaryngology Head and Neck Surgery, Chinese PLA General Hospital enrolled in the study, from June 2015 to September 2021. Trio WES was performed in 95 cases and their parents, trio/quadro NGS in 2009 cases and their family members, and singleton NGS in 159 cases.

And 81 cases from 74 families with detected *MYO15A* variants, related to NSHL, were analyzed for the assessment of the correlation between the *MYO15A* genotype and the NSHL phenotype. Detailed interviews were conducted with probands and their families to obtain their medical and familial histories.

All underwent testing that included physical examination, otoscopy, pure tone audiometry (PTA), tympanometry, assessment of auditory brainstem responses (ABR), distortion product otoacoustic emission (DPOAE), multiple auditory steady-state evoked responses (ASSR), temporal bone computerized tomography scans, and magnetic resonance of the brain. The definition for the severity of hearing impairment, according to pure-tone audiometry (PTA) of the better ear, was made based on the average hearing threshold level at four frequencies (500, 1000, 2000 and 4000 Hz) of air conduction. 26–40 dB HL were considered to be mild hearing loss; 41–55 dB HL, moderate hearing loss; 56–70 dB, moderately severe hearing loss; 71–90 dB HL, severe hearing loss; > 90 dB HL, profound hearing loss. The occurrence of hearing loss was categorized as prelingual (≤ 3 years) or post-lingual (> 3 years). Asymmetric hearing loss (AHL) was defined as greater than 15 dB between the ears at 0.5, 1, and 2 kHz or greater than 20 dB at 4 kHz on the audiogram (American Academy Otolaryngology-Head Neck Surgery 1997) [14] as reported previously [15].

Peripheral blood samples were collected from all cases, their parents and siblings (if any). All cases obtained informed consent for the performed molecular genetic analysis and their clinical data publication. The study was approved by the Ethics Committee of the Chinese PLA General Hospital (reference number S2016-120–02). Written informed consent was obtained from the participants and in the case of young cases from their parents.

### Targeted deafness gene capture and NGS

Targeted deafness gene capture and NGS were performed as previously reported [16]. DNA samples of 64 cases from 58 families were subjected to targeted NGS, 35 cases of them conducted trio (proband and parents) targeted NGS and 29 cases conducted quarto (proband, parents and sibling) targeted NGS. The proband received the panel test containing 168 deafness-related genes (Additional files 1: Table S1). All coding exons, along with 100-bp flanking regions were sequenced on the Illumina HiSeq 2000 (Illumina, San Diego, CA, USA) using the MyGenostics gene enrichment system (MyGenostics, Boston, MA, USA).

### Whole-exome sequencing (WES)

Illumina NovaSeq6000 sequencing platform was used to conduct the WES by MyGenostics (Beijing, China) (detailed procedures shown in Additional files 3). DNA samples from 17 *MYO15A*-related cases and their parents were subjected to trio WES and subsequently validated by Sanger sequencing. The nomenclature of the mutation described in Table 1 is based on *MYO15A* cDNA and protein accession numbers NM_016239.3 and NP_057323.3, respectively. We used the genomic coordinates from GRCH37/hg19 constructed from the human genome.

### Bioinformatics

After sequencing the targeted region, quality control was performed to ensure the accuracy of the data. Low-quality data were filtered out to obtain clean sequencing data. Burrows-Wheeler alignment was used to align the clean sequence to the human reference genome hg19. Genome Analysis Toolkit (GATK) was used to detect single-nucleotide and insertion/deletion polymorphisms (indel). The NCBI ClinVar (https://www.ncbi.nlm.nih.gov/clinvar/, last accessed date 16 December 2021), the Human Gene Mutation Database (HGMD, http://www.hgmd.cf.ac.uk/ac/, last accessed date 16 December 2021), the Genome Aggregation Database (gnomAD, https://gnomad.broadinstitute.org, last accessed date 16 December 2021) and the Deafness Variation Database (DVD v8, https://deafnessvariationdatabase.org) were used to obtain the variants information, including gene information, variant consequence, minor allele frequency (MAF), altered protein function, and related disease information. The predictive score of pathogenicity of the variation was calculated, and the effect of amino acid substitution on protein structure and function was evaluated by Polyphen2 (http://genetics.bwh.harvard.edu/pph2/) and SIFT (http://provean.jcvi.org/). Pathogenicity was assessed according to the expert specification of the American Society for Medical Genetics and Genomics/ Association of Medical Pathology (ACMG/AMP) guidelines for genetic HL[17].

### Sanger sequencing

Presumed pathogenic or likely pathogenic variants and variants of uncertain significance detected by WES and deafness gene panel in the probands were subsequently validated by a polymerase chain reaction (PCR) amplification and Sanger sequencing. Segregation analysis was performed on the probands and their family members. The primer sets are listed in Additional files 2: Table S2.

## Results

### Detected variants

Clinical features and genotypes of the pathogenic, likely pathogenic and uncertain *MYO15A* variants are summarized in Table 1. In particular, 4 cases were found to carry homozygous variants, 77 carried compound heterozygous variants.

**Table 1:** Summary of the *MYO15A* variants identified in this study

Nucleotide change	Protein change	Exon	Number of patient	Hearing level	Variant type	Criteria for pathogenicity	ACMG classification	MAF (gnomAD in east Asian)	MAF (gnomAD in total)	References
c.198_199delCC	p.Gln68Glufs*158	2	1	Severe	Frameshift	PVS1_Very Strong,PM2_Moderate,	LP	NA	NA	
c.220_221delAG	p.Arg74Glufs*153	2	1	Profound	Frameshift	PVS1_Very Strong,PM2_Moderate,PP3_Supporting	P	NA	NA	
c.596C > G	p.Ser199Ter	2	1	Severe	Nonsense	PVS1_Very Strong,PM2_Moderate,PP3_Supporting	P	NA	NA	
c.735C > G	p.Tyr245Ter	2	1	Profound	Nonsense	PVS1_Very Strong,PM2_Moderate,PP3_Supporting	P	NA	NA	
c.900delT	p.Pro301Argfs*142	2	1	Profound	Frameshift	PVS1_Very Strong,PM2_Moderate	LP	NA	NA	
c.1101del	p.Tyr368Thrfs*76	2	1	Moderately severe	Frameshift	PVS1_Very Strong,PM2_Moderate	LP	NA	NA	
c.1179insC	p.Glu396Argfs*36	2	2	Mild to profound	Frameshift	PVS1_Very Strong,PP5_Strong,PP4_Moderate	P	0.0000643	0.000334	Bashir (2012)
c.1185dupC	p.Glu396Argfs*36	2	1	Profound	Frameshift	PVS1_Very Strong,PP5_Strong,PM2_Moderate	P	0.000334	0.0000643	Bashir (2012), Miyagawa (2013)
c.1201delT	p.Tyr401Thrfs*43	2	1	Moderately severe	Frameshift	PVS1_Very Strong,PM2_Moderate	LP	NA	NA	
c.1261C > T	p.Pro421Ser	2	1	Mild	Missense	PM2_Supporting,BP4_Supporting	U	0.000167	0.0000121	
c.1651G > A	p.Ala551Thr	2	1	Severe	Missense	PM2_Supporting,BP4_Supporting	U	0.000358	0.0000269	
c.2231C > A	p.Ser744Ter	2	1	Profound	Nonsense	PVS1_Very Strong,PM2_Moderate	LP	NA	NA	
c.2957delC	p.Thr986Ter	2	1	Moderate	Frameshift	PVS1_Very Strong,PM2_Moderate	LP	NA	NA	Nal (2007)
c.3118delC	p.Lys1042Argfs*16	2	1	Profound	Frameshift	PVS1_Very Strong,PM2_Moderate	LP	0.0000556	0.00000402	
c.3136delC	p.Lys1048Argfs*10	2	1	Severe	Frameshift	PVS1_Very Strong,PM2_Moderate	LP	NA	NA	
c.3354G > T	p.Met1118Ile	2	1	Severe	Frameshift	PM2_Supporting,PP3_Supporting	U	NA	NA	
c.3524dupA	p.Ser1176Valfs*13	2	3	Moderate to profound	Frameshift	PVS1_Very Strong,PP5_Strong,PM2_Moderate	P	0.00195	0.000142	Li (2016)
c.3602G > A	p.Arg1201Gln	2	2	Moderately severe to profound	Missense	PM2_Supporting,BP4_Supporting	U	–	0.0000164	
c.3700C > T	p.Gln1234Ter	4	1	Profound	Nonsense	PVS1_Very Strong,PM2_Moderate,PP3_Supporting	P	NA	NA	
c.3829C > T	p.Gln1277Ter	5	1	Profound	Nonsense	PVS1_Very Strong,PM2_Moderate,PP3_Supporting	P	NA	NA	
c.3866 + 1G > A	splicing	Intron 5	2	Profound	Nonsense	PVS1_Very Strong,PM2_Moderate,PP5_Moderate,PP3_Supporting	P	–	0.0000161	Nal (2007),Naz (2017)
c.3926A > T	p.Gln1309Leu	6	1	Profound	Missense	PM2_Strong,PP3_Supporting	U	0.0000556	0.00000401	
c.3971C > A	p.Ala1324Asp	7	2	Profound	Missense	PP5_Strong,PM2_Moderate,PP3_Supporting	LP	0.0000556	0.00000401	
c.4037A > G	p.Lys1346Arg	8	2	Profound	Missense	PVS1_Very Strong,PM2_Supporting	U	NA	NA	
c.4198G > A	p.Val1400Met	10	1	Moderately severe	Missense	PP5_Very Strong,PM2_Moderate,PP3_Supporting	P	0.0000556	0.0000361	Manzoli (2016),Cengiz (2010)
c.4252G > A	p.Gly1418Arg	11	1	Profound	Missense	PM2_Strong,PP5_Moderate,PP3_Supporting	LP	–	0.00000803	Park (2014)
c.4310A > G	p.Tyr1437Cys	11	1	Profound	Missense	PM2_Strong,PP5_Moderate,PP3_Supporting	LP	–	0.0000122	Sloan-Heggen (2016)
c.4322G > T	p.Gly1441Val	11	1	Severe	Missense	PP5_Very Strong,PM2_Strong,PP3_Supporting	P			
c.4430G > A	p.Arg1477His	12	1	Moderately severe	Frameshift	PM2_Moderate,PP3_Supporting	U	–	0.0000361	
c.4441 T > C	p.Ser1481Pro	13	4	Profound	Missense	PM2_Moderate,PP3_Supporting	U	0.0000556	0.00000401	Cengiz (2010), Diaz-Horta (2012)
c.4519C > T	p.Arg1507Ter	13	1	Profound	Missense	PVS1_Very Strong,PM2_Moderate,PP3_Supporting,PP5_Supporting	P	–	0.00000401	
c.4567C > A	p.Leu1523Met	13	1	Moderately severe	Missense	PM2_Moderate,PP3_Supporting	U	NA	NA	
c.4596 + 1G > A	splicing	Intron 13	1	Profound	Splicing	PVS1_Very Strong,PM2_Moderate,PP5_Moderate,PP3_Supporting	P	–	0.0000122	
c.4642G > A	p.Ala1548Thr	14	1	Profound	Missense	PM2_Moderate,PP3_Supporting	U	–	0.0000201	Atik (2015)
c.4676 T > C	p.Leu1559Ser	15	1	Profound	Missense	PM2_Moderate,PP3_Supporting	U	–	0.00000401	
c.4777G > A	p.Glu1593Lys	15	2	Profound	Missense	PM2_Strong,PP3_Strong	P	–	0.0000656	Sloan-Heggen (2016)
c.4784 T > C	p.Leu1595Pro	15	1	Profound	Missense	PM2_Moderate,PP3_Supporting	U	–	0.00000401	
c.4793A > G	p.Asn1598Ser	16	1	Profound	Missense	PM2_Strong,PP3_Supporting	U	NA	NA	
c.4817A > G	p.Asn1606Ser	16	2	Profound	Missense	PM2_Strong,PP3_Supporting	U	NA	NA	
c.4898 T > C	p.Ile1633Thr	17	4	Moderate to profound	Missense	PM2_Moderate,PP3_Supporting	U	0.000111	0.00000805	Gu (2015);Rehman (2016)
c.4987G > A	p.Asp1663Asn	17	1	Severe	Missense	PM2_Strong,PP3_Supporting	U	–	0.0000161	
c.5036G > A	p.Cys1679Tyr	18	1	Profound	Missense	PM2_Strong,PP3_Supporting	U	NA	NA	
c.5134-1G > A	splicing	Intron 18	1	Profound	Splicing	PVS1_Very Strong,PM2_Moderate,PP3_Supporting	P	NA	NA	
c.5360G > A	p.Arg1787Lys	20	1	Profound	Missense	PVS1_Very Strong,PM2_Moderate	LP	NA	NA	
c.5362 T > G	p.Cys1788Gly	20	1	Severe	Missense	PVS1_Very Strong,PM2_Supporting,PP3_Supporting	P	NA	NA	
c.5504G > T	p.Arg1835Leu	21	1	Severe	Missense	PM2_Strong,PP3_Supporting	U	–	–	
c.5507 T > C	p.Leu1836Pro	21	1	Profound	Missense	PM2_Moderate,PP3_Supporting	U	NA	NA	
c.5722_5725del	p.Thr1908Cysfs*40	24	1	Moderately severe	Frameshift	PVS1_Very Strong,PM2_Moderate,PP3_Supporting	P	NA	NA	
c.5809C > G	p.Arg1937Gly	24	1	Profound	Missense	PM2_Moderate,PP3_Supporting	U	NA	NA	Sloan-Heggen (2016),Fattahi (2012)
c.5835 T > G	p.Tyr1945Ter	24	1	Profound	Nonsense	PVS1_Very Strong,PM2_Moderate,PP5_Moderate,PP3_Supporting	P	NA	NA	Chang (2015)
c.5964 + 3G > A	-	Intron 26	3	Profound	Non coding	PM2_Moderate,BP4_Supporting	U	0.000391	0.0000287	Gao (2013)
c.5977C > T	p.Arg1993Trp	27	1	Profound	Missense	PM5_Moderate,PM2_Supporting,PP3_Supporting	U	0.000125	0.0000321	
c.6177 + 1G > T	splicing	Intron 28	3	Profound	Splicing	PVS1_Very Strong,PM2_Moderate,PP3_Supporting,PP5_Supporting	P	NA	NA	
c.6338 T > A	p.Ile2113Asn	30	2	Profound	Missense	PM1_Moderate,PM2_Moderate,PM5_Moderate,PP3_Supporting	LP	NA	NA	
c.6442 T > A	p.Trp2148Arg	30	1	Profound	Missense	PP5_Strong,PM1_Moderate,PM2_Moderate,PP3_Supporting	LP	NA	NA	
c.6510-1G > T	splicing	Intron 30	1	Profound	Splicing	PVS1_Very Strong,PM2_Moderate,PP3_Supporting,PP5_Supporting	P	NA	NA	
c.6611G > A	p.Arg2204His	31	1	Profound	Missense	PM2_Strong,PM1_Moderate,PM5_Moderate,PP3_Supporting	LP	NA	NA	
c.6616 T > A	p.Leu2206Ile	31	1	Profound	Missense	PM1_Moderate, PM2_Moderate, BP4_Supporting	U	NA	NA	
c.6620C > T	p.Pro2207Leu	31	1	Profound	Missense	PM1_Moderate, PM2_Moderate, PP3_Supporting	U	NA	NA	
c.6634G > A	p.Glu2212Lys	31	1	Profound	Missense	PM2_Strong, PM1_Moderate, PP3_Supporting	LP	–	0.0000241	
c.6716A > C	p.His2239Pro	31	1	Profound	Missense	PM2_Strong, PP3_Supporting	U	NA	NA	
c.6764 + 1G > T	splicing	Intron 32	1	Profound	Splicing	PVS1_Very Strong, PM2_Moderate, PP3_Supporting	P	NA	NA	
c.6956 + 9C > G	-	33	2	Profound	Non coding	PM2_Moderate, BP4_Supporting	U	0.0000706	0.00000535	Yang (2013)
c.7396-1G > A	splicing	Intron 37	2	Profound	Splicing	PVS1_Very Strong, PP5_Very Strong, PM2_Moderate, PP3_Supporting	P	0.000192	0.0000141	
c.7519delC	p.Pro2508Leufs*35	39	1	Moderate	Frameshift	PVS1_Very Strong, PM2_Moderate	LP	NA	NA	
c.7698_7699delTG	p.Glu2567Alafs*25	40	1	Severe	Frameshift	PVS1_Very Strong, PM2_Moderate, PP3_Supporting	P	NA	NA	
c.7770delC	p.Arg2591Glyfs*14	40	2	Profound	Frameshift	PVS1_Very Strong, PM2_Moderate	LP	NA	NA	
c.8129insT	p.Asp2711fs*1	43	1	Severe	Nonsense	PVS1_Very Strong, PM2_Moderate, PP3_Supporting	P	NA	NA	
c.8151delC	p.Leu2718Cysfs*20	45	1	Profound	Frameshift	PVS1_Very Strong, PM2_Moderate	LP	NA	NA	
c.8240_8241delAC	p.Gln2749Glufs*93	45	1	Profound	Frameshift	PVS1_Very Strong, PM2_Moderate, PP3_Supporting	P	NA	NA	
c.8283_8306delGGTCAGCACTGCACGAGACACCTG	p.2761_2769del	45	1	Profound	In frame	PM2_Moderate, PM4_Moderate, PP3_Supporting	U	NA	NA	
c.8324G > T	p. Arg2775Leu	46	2	Profound	Missense	PM2_Strong, PP3_Supporting	U	NA	NA	
c.8324G > A	p. Arg2775His	46	1	Profound	Missense	PM2_Strong, PP3_Supporting	U	0.0000557	0.00000804	Yang (2013);Sloan-Heggen (2016)
c.8340G > A	p.Thr2780Thr	46	2	Profound	Synonymous	PVS1_Very Strong, PM2_Moderate, PP5_Supporting	P	–	0.00000803	Danial-Farran (2018)
c.8362C > T	p.Gln2788Ter	46	1	Profound	Nonsense	PVS1_Very Strong, PM2_Moderate, PP3_Supporting	P	NA	NA	
c.8458A > C	p.Ser2820Arg	46	2	Profound	Missense	PVS1_Very Strong, PM2_Moderate, PP3_Supporting	P	NA	NA	
c.8459G > C	p.Ser2820Thr	47	1	Profound	Missense	PVS1_Very Strong, PM2_Moderate	LP	NA	NA	
c.8713 + 1delGTCA	splicing	Intron 49	1	Severe	Splicing	PVS1_Very Strong, PM2_Moderate, PP3_Supporting	P	NA	NA	
c.8745_8747delGGT	p.2915_2916del	50	1	Profound	In frame	PM2_Moderate, PM4_Moderate, PP3_Supporting	U	NA	NA	
c.8791delT	p.Trp2931Glyfs*103	51	1	Profound	Frameshift	PVS1_Very Strong, PM2_Moderate, PP3_Supporting	P	NA	NA	
c.8827insT	p.Ser2945Phefs*55	51	3	Profound	Frameshift	PVS1_Very Strong, PM2_Moderate, PP3_Supporting	P	0.000113	0.00000837	
c.8828 T > C	p.Phe2943Ser	51	1	Profound	Missense	PM2_Moderate, PP3_Supporting	U	NA	NA	
c.8976insA	p.Val2993Serfs*7	52	1	Profound	Frameshift	PVS1_Very Strong, PM2_Moderate	LP	NA	NA	
c.9358C > T	p.Gln3120Ter	56	2	Severe to profound	Nonsense	PVS1_Very Strong, PM2_Moderate, PP3_Supporting	P	0.0000556	0.00000402	
c.9400C > T	p.Arg3134Ter	57	1	Severe	Nonsense	PVS1_Very Strong, PM2_Moderate, PP5_Moderate, PP3_Supporting	P	–	0.00000401	
c.9401G > C	p.Arg3134Pro	57	1	Profound	Missense	PM2_Moderate, PP3_Supporting	U	NA	NA	
c.9478C > T	p.Leu3160Phe	57	2	Moderate to severe	Missense	PP3_Supporting, BS2_Strong	U	0.00289	0.00691	Nal (2007),Miyagawa (2013)
c.9532 T > C	p.Cys3178Arg	58	2	Profound	Missense	PM2_Moderate, PP3_Supporting	U	NA	NA	
c.9534C > A	p.Cys3178Ter	58	1	Profound	Nonsense	PVS1_Very Strong, PM2_Moderate, PP3_Supporting	P	NA	NA	
c.9690 + 1G > A	splicing	Intron 59	3	Profound	Splicing	PVS1_Very Strong, PP5_Strong, PM2_Moderate, PP3_Supporting	P	NA	NA	Chen (2015)
c.9787 + 1G > A	splicing	Intron 60	1	Profound	Splicing	PVS1_Very Strong, PM2_Moderate, PP3_Supporting	P	NA	NA	
c.9941delA	p.Tyr3314Serfs*9	61	1	Profound	Frameshift	PVS1_Very Strong, PM2_Moderate, PP3_Supporting	P	NA	NA	
c.9942_9943delCAinsTGTGTG	p.Tyr3314Ter	61	1	Profound	Nonsense	PVS1_Very Strong, PM2_Moderate, PP3_Supporting	P	NA	NA	
c.10129dup	p.Ala3377Glyfs*75	63	1	Moderately severe	Frameshift	PVS1_Very Strong, PM2_Moderate	LP	NA	NA	
c.10177C > T	p.Gln3393Ter	63	1	Severe	Nonsense	PVS1_Very Strong, PM2_Moderate, PP3_Supporting	P	NA	NA	
c.10183C > T	p.Leu3395Phe	63	1	Profound	Missense	PM2_Supporting, PP3_Supporting	U	NA	NA	
c.10245_10247delCTC	p.3415_3416del	64	12	Profound	Frameshift	PM2_Moderate, PM4_Moderate, PP3_Supporting, PP5_Supporting	LP	0.000389	0.0000281	Chang (2018), Miyagawa (2015)
c.10250_10252del	p.Ser3417del	64	2	Profound	Frameshift	PM2_Moderate, PM4_Moderate, PP3_Supporting, PP5_Supporting	LP	0.000389	0.0000281	
c.10251_10253delCTT	p.3417_3418del	64	7	Severe to profound	In frame	PM2_Moderate, PP3_Supporting	U	0.000111	0.000016	Yang (2013)
c.10291_10305delGCCCCTTGCATCCTT	p.3431_3435delAlaProCysIleLeu	64	1	Profound	In frame	PM2_Moderate, PM4_Moderate, PP3_Supporting	U	NA	NA	
c.10350 + 2 T > G	splicing	Intron 64	1	Profound	Splicing	PVS1_Very Strong, PM2_Moderate, PP3_Supporting	P	0.0000556	0.00000401	
c.10419_10423delCAGCT	p.Ser3474Profs*42	65	11	Profound	Frameshift	PVS1_Very Strong, PM2_Moderate, PP3_Supporting	P	NA	NA	

^a^P pathogenic, LP likely pathogenic, U uncertain significance

In our study we have found 102 *MYO15A* variants, among which the most recurrent variants were c.10245_10247delCTC (0.27%, 12/4526), followed by c.10419_10423delCAGCT (0.24%, 11/4526), c.10251_10253delCTT (0.15%, 7/4526), c.4441 T > C (0.09%, 4/4526), c.4898 T > C (0.09%, 4/4526), c.3524dupA (0.07%, 3/4526), c.5964 + 3G > A (0.07%, 3/4526), c.6177 + 1G > T(0.07%, 3/4526), c.8827insT (0.07%, 3/4526) and c.9690 + 1G > A (0.07%, 3/4526). Other variants appeared only once or twice (Table 1).

**Fig. 1:**
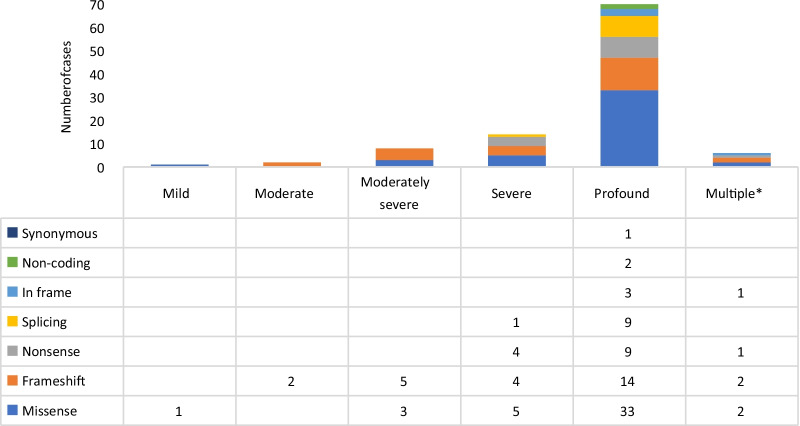
The degree of HL and the types of detected variants in the identified *MYO15A* variations. *The Multiple column represented the cases with the same variations showed different degrees of HL

Our analysis showed that the most common type of *MYO15A* variants was missense (44/102, 43.14%), followed by frameshift (27/102, 26.47%), nonsense (14/102, 13.72%), splice site (10/102, 9.80%), in frame (4/102, 3.92%), non-coding (2/102, 1.96%) and synonymous (1/102, 0.98%) (Fig. 1). The variants showed the various degree of HL, although the cases with the same variant type showed different phenotypes. In frame and splice variants showed more possibilities to cause profound HL, and frameshift and missense variants related to various degrees of HL (Fig. 1).

**Fig. 2:**
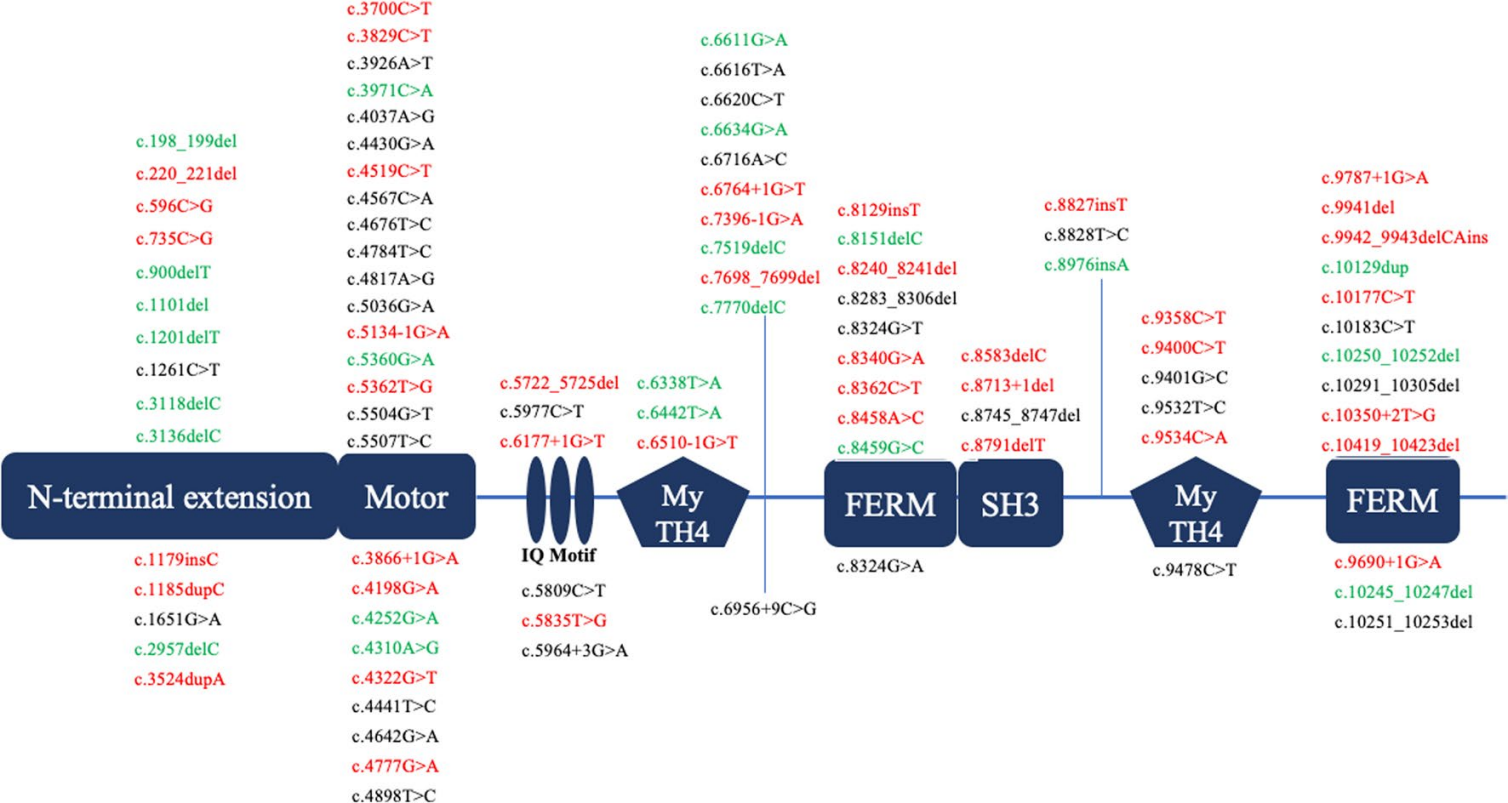
The locations of the detected 102 *MYO15A* variants. The figure shows the locations of 102 *MYO15A* variants correlated with NSHL found in this study. The previously reported ones are shown at the bottom. Pathogenic variants were expressed in red words, likely pathogenic variants in green words, and VUS in black words

The variants were located in 41 of the 66 protein-coding exons of the *MYO15A* gene (Table 1) and identified in all domains in this study. Seventy-eight novel and 24 reported variants were identified, and all of them were confirmed by Sanger sequencing. (Fig. 2).

According to the guidelines of the ACMG/AMP on hereditary hearing loss, the variations in the *MYO15A* were manually classified [17, 18]. Based on the ACMG/AMP rating, ClinVar, HGVS and DVD database, respectively, the pathogenicity of the 102 *MYO15A* variants identified in this study included 40 pathogenic (P), 24 likely pathogenic (LP) and 38 variants with uncertain significance (VUS). (Table 2) We identified 36 cases with bi-allelic *MYO15A* pathogenic or likely pathogenic variants. The others with VUS in one of the alleles (LP/VUS, P/VUS and VUS/VUS) were also included in the study that classified as the best candidate of DFNB3. We also compared the severity of HL by the pathogenicity of variants. The results were inconclusive, and even the cases with the same variations showed various phenotypes (Table 3).

**Table 2:** Summary of the genotype–phenotype of patients in the *MYO15A* identified in this study

Patient Number	Sex^a^	Ethnicity	Age of visiting(yo)	Age of Onset(yo)	Variant 1	Variant 2	Variant type	Variant Classification^b^	Truncating^c^	Degree of HL	Audiogram Configuration
M3	F	Han	7	0	c.4777G > A(p.Glu1593Lys)	c.8745_8747delGGT(p.2915_2916del)	Compound heterozygous	P/U	0/1	Profound	Down-sloping
M23	M	Han	2	0	c.5504G > T(p.Arg1835Leu)	c.10251_10253delCTT(p.3417_3418del)	Compound heterozygous	U/U	0/1	Severe	Flat
M73	F	Han	1	0	c.8713 + 1delGTCA(splicing)	c.9400C > T(p.Arg3134Ter)	Compound heterozygous	P/P	1/0	Severe	Undefined
M80	M	Han	44	41	c.2957delC(p.Thr986Ter)	c.9478C > T(p.Leu3160Phe)	Compound heterozygous	LP/U	1/0	L:Severe; R:Moderate	Undefined
M113	M	Han	26	0	c.8459G > C(p.Ser2820Thr)	c.10245_10247delCTC(p.3415_3416del)	Compound heterozygous	LP/LP	0/1	Profound	Total deafness
M207	M	Han	3	0	c.10245_10247delCTC(p.3415_3416del)	c.10251_10253delCTT(p.3417_3418del)	Compound heterozygous	LP/U	1/1	Profound	Down-sloping
M247	F	Han	25	0	c.5977C > T(p.Arg1993Trp)	c.10245_10247delCTC(p.3415_3416del)	Compound heterozygous	U/LP	0/1	Profound	Down-sloping
M251	F	Han	1	0	c.5964 + 3G > A	c.8828 T > C(p.Phe2943Ser)	Compound heterozygous	U/U	1/0	Profound	Total deafness
M291	M	Han	12	0	c.3926A > T(p.Gln1309Leu)	c.8827insT(p.Ser2945Phefs*55)	Compound heterozygous	U/P	0/1	Profound	Total deafness
M294	M	Han	27	0	c.8791delT(p.Trp2931Glyfs*103)	c.10245_10247delCTC(p.3415_3416del)	Compound heterozygous	P/LP	1/1	Profound	Total deafness
M337	M	Han	7	0	c.5362 T > G(p.Cys1788Gly)	c.8129insT(p.Asp2711fs*1)	Compound heterozygous	P/P	0/1	Severe	Flat
M373	F	Han	3	0	c.8976insA(p.Val2993Serfs*7)	c.9942_9943delCAinsTGTGTG(p.Tyr3314Ter)	Compound heterozygous	LP/P	1/1	Profound	Total deafness
M445	F	Han	2	0	c.10251_10253delCTT(p.3417_3418del)	c.10419_10423delCAGCT(p.Ser3474Profs*42)	Compound heterozygous	U/P	1/1	Profound	Undefined
M448	M	Han	27	0	c.7396-1G > A(splicing)	c.8827insT(p.Ser2945Phefs*55)	Compound heterozygous	P/P	1/1	Profound	Total deafness
M448-5	M	Han	30	0	c.7396-1G > A(splicing)	c.8827insT(p.Ser2945Phefs*55)	Compound heterozygous	P/P	1/1	Profound	Total deafness
M488	F	Han	0	0	c.8340G > A(p.Thr2780Thr)	c.9532 T > C(p.Cys3178Arg)	Compound heterozygous	P/U	1/0	Profound	Total deafness
M488-1	M	Han	31	0	c.8340G > A(p.Thr2780Thr)	c.8340G > A(p.Thr2780Thr)	Homozygous	P/P	1/1	Profound	Total deafness
M488-2	F	Han	33	1	c.3971C > A(p.Ala1324Asp)	c.9532 T > C(p.Cys3178Arg)	Compound heterozygous	LP/U	0/0	Profound	Total deafness
M492	F	Han	3	0	c.5964 + 3G > A	c.6764 + 1G > T(splicing)	Compound heterozygous	U/P	1/1	Profound	Total deafness
M494	F	Han	5	0	c.9358C > T(p.Gln3120Ter)	c.10419_10423delCAGCT(p.Ser3474Profs*42)	Compound heterozygous	P/P	1/1	Profound	Total deafness
M544	F	Han	21	0	c.6177 + 1G > T(splicing)	c.8458A > C(p.Ser2820Arg)	Compound heterozygous	P/P	1/0	Profound	Total deafness
M544-3	F	Han	24	0	c.6177 + 1G > T(splicing)	c.8458A > C(p.Ser2820Arg)	Compound heterozygous	P/P	1/0	Profound	Total deafness
M613	F	Han	15	0	c.3118delC(p.Lys1042Argfs*16)	c.10245_10247delCTC(p.3415_3416del)	Compound heterozygous	LP/LP	1/1	Profound	Undefined
M623	M	Han	6	3	c.10251_10253delCTT(p.3417_3418del)	c.10251_10253delCTT(p.3417_3418del)	Homozygous	U/U	1/1	Severe	Total deafness
M623-3	M	Han	8	3	c.10251_10253delCTT(p.3417_3418del)	c.10251_10253delCTT(p.3417_3418del)	Homozygous	U/U	1/1	Profound	Total deafness
M627	M	Han	3	0	c.5507 T > C(p.Leu1836Pro)	c.5835 T > G(p.Tyr1945Ter)	Compound heterozygous	U/P	0/1	Profound	Total deafness
M646	M	Han	7	4	c.1179insC(p.Glu396Argfs*36)	c.1261C > T(p.Pro421Ser)	Compound heterozygous	P/U	1/0	L:Profound; R:Mild	Undefined
M653	F	Han	5	0	c.8283_8306delGGTCAGCACTGCACGAGACACCTG(p.2761_2769del)	c.10245_10247delCTC(p.3415_3416del)	Compound heterozygous	U/LP	1/1	Profound	Total deafness
M656	M	Han	6	0	c.6956 + 9C > G	c.10419_10423delCAGCT(p.Ser3474Profs*42)	Compound heterozygous	U/P	1/1	Profound	Total deafness
M659	M	Han	6	0	c.6177 + 1G > T(splicing)	c.9690 + 1G > A(splicing)	Compound heterozygous	P/P	1/1	Profound	Total deafness
M678	F	Tujia	1	0	c.8324G > T(p.Arg2775Leu)	c.10419_10423delCAGCT(p.Ser3474Profs*42)	Compound heterozygous	U/P	0/1	Profound	Total deafness
M722	M	Han	2	0	c.6716A > C(p.His2239Pro)	c.9787 + 1G > A(splicing)	Compound heterozygous	U/P	0/1	Profound	Total deafness
M766	M	Han	4	0	c.6620C > T(p.Pro2207Leu)	c.10245_10247delCTC(p.3415_3416del)	Compound heterozygous	U/LP	0/1	Profound	Total deafness
Y770	M	Han	5	1	c.10250_10252delGCT(p.3417delSer)	c.10419_10423delCAGCT(p.Ser3474Profs*42)	Compound heterozygous	LP/P	1/1	Profound	Total deafness
M771	F	Han	8	0	c.3524dupA(p.Ser1176Valfs*13)	c.4441 T > C(p.Ser1481Pro)	Compound heterozygous	P/P	1/0	Profound	Total deafness
M817	M	Han	26	0	c.4519C > T(p.Arg1507Ter)	c.5964 + 3G > A	Compound heterozygous	P/U	1/0	Profound	Total deafness
Y840	F	Han	22	0	c.4898 T > C (p.Ile1633Thr)	c.6338 T > A (p.Ile2113Asn)	Compound heterozygous	U/LP	0/0	Profound	Total deafness
Y840-3	M	Han	20	0	c.4898 T > C (p.Ile1633Thr)	c.6338 T > A (p.Ile2113Asn)	Compound heterozygous	U/LP	0/0	Profound	Total deafness
M880	M	Han	11	0	c.10245_10247delCTC(p.3415_3416del)	c.10245_10247delCTC(p.3415_3416del)	Homozygous	LP/LP	1/1	Profound	Flat
Y885	F	Han	8	0	c.4777G > A(p.Glu1593Lys)	c.5809C > G (p.Arg1937Gly)	Compound heterozygous	P/U	0/0	Profound	Total deafness
Y914	M	Han	7	1	c.4784 T > C (p.Leu1595Pro)	c.6956 + 9C > G	Compound heterozygous	U/U	0/1	Profound	Total deafness
M930	M	Han	8	0	c.3866 + 1G > A(splicing)	c.8240_8241delAC(p.Gln2749Glufs*93)	Compound heterozygous	P/P	1/1	Profound	Total deafness
M1039	M	Han	3	0	c.4037A > G(p.Lys1346Arg)	c.10419_10423delCAGCT(p.Ser3474Profs*42)	Compound heterozygous	U/P	0/1	Profound	Total deafness
M1058	F	Han	2	0	c.3866 + 1G > A(splicing)	c.3971C > A(p.Ala1324Asp)	Compound heterozygous	P/LP	1/0	Profound	Total deafness
M1125	F	Han	3	0	c.8362C > T(p.Gln2788Ter)	c.10251_10253delCTT(p.3417_3418del)	Compound heterozygous	P/U	1/1	Profound	Total deafness
M1197	M	Han	6	0	c.9534C > A(p.Cys3178Ter)	c.10245_10247delCTC(p.3415_3416del)	Compound heterozygous	P/LP	1/1	Profound	Total deafness
M1207	M	Han	6	0	c.735C > G(p.Tyr245Ter)	c.10419_10423delCAGCT(p.Ser3474Profs*42)	Compound heterozygous	P/P	1/1	Profound	Total deafness
M1247	M	Han	30	0	c.4322G > T(p.Glu1441Val)	c.10251_10253delCTT(p.3417_3418del)	Compound heterozygous	P/U	0/1	Severe	Undefined
					c.1651G > A(p.Ala551Thr)		Compound heterozygous	U/U	0/1		
M1324	F	Han	36	0	c.9401G > C(p.Arg3134Pro)	c.10245_10247delCTC(p.3415_3416del)	Compound heterozygous	U/LP	0/1	Profound	Undefined
Y1457	F	Han	5	0	c.1201delT(p.Tyr401Thrfs*43)	c.5722_5725delA(p.Thr1908Cysfs*40)	Compound heterozygous	LP/P	1/1	Moderately severe	Down-sloping
YL1467	M	Han	10	4	c.3602G > A(p.Arg1201Gln)	c.4567C > A(p.Leu1523Met)	Compound heterozygous	U/U	0/0	Moderately severe	Down-sloping
M1550	M	Han	6	0	c.596C > G(p.Ser199Ter)	c.10177C > T(p.Gln3393Ter)	Compound heterozygous	P/P	1/1	Severe	Down-sloping
					c.3354G > T(p.Met1118Ile)		Compound heterozygous	U/P	0/1		
M1584	M	Han	8	0	c.10245_10247delCTC(p.3415_3416del)	c.10251_10253delCTT(p.3417_3418del)	Compound heterozygous	LP/U	1/1	Profound	Total deafness
M1586	F	Han	2	0	c.198_199delCC(p.Gln68Glufs*158)	c.7698_7699delTG(p.Glu2567Alafs*25)	Compound heterozygous	LP/P	1/1	Severe	Undefined
M1611	F	Korean	28	8	c.3602G > A(p.Arg1201Gln)	c.10350 + 2 T > G(splicing)	Compound heterozygous	LP/P	0/1	Profound	Undefined
					c.900delT(p.Pro301Argfs*142)		Compound heterozygous	LP/P	1/1		
M1671	F	Han	5	0	c.10245_10247delCTC(p.3415_3416del)	c.10419_10423delCAGCT(p.Ser3474Profs*42)	Compound heterozygous	LP/P	1/1	Profound	Total deafness
YL1728	M	Han	3	0	c.1101del(p.Tyr368Thrfs*76)	c.10129dup(p.Ala3377Glyfs*75)	Compound heterozygous	LP/LP	1/1	Moderately severe	Down-sloping
M1802	M	Han	28	2	c.4898 T > C (p.Ile1633Thr)	c.10419_10423delCAGCT(p.Ser3474Profs*42)	Compound heterozygous	U/P	0/1	Profound	Undefined
M1878	M	Han	7	0	c.4817A > G(p.Asn1606Ser)	c.7770delC(p.Arg2591Glyfs*14)	Compound heterozygous	U/LP	0/1	Profound	Total deafness
M1878-2	F	Han	32	0	c.4817A > G(p.Asn1606Ser)	c.6616 T > A(p.Leu2206Ile)	Compound heterozygous	U/U	0/0	Profound	Undefined
M1879	M	Han	2	0	c.6634G > A(p.Glu2212Lys)	c.10419_10423delCAGCT(p.Ser3474Profs*42)	Compound heterozygous	LP/P	0/1	Profound	Total deafness
M1928	M	Han	13	10	c.4198G > A(p.Val1400Met)	c.4430G > A (p.Arg1477His)	Compound heterozygous	P/U	0/0	Moderately severe	Down-sloping
M1959	F	Han	10	2	c.6442 T > A(p.Trp2148Arg)	c.10183C > T (p.Leu3395Phe)	Compound heterozygous	LP/U	0/0	Profound	Total deafness
M1960	F	Han	5	0	c.4252G > A(p.Gly1418Arg)	c.4441 T > C(p.Ser1481Pro)	Compound heterozygous	LP/U	0/0	Profound	Total deafness
M1997	F	Han	8	7	c.4898 T > C (p.Ile1633Thr)	c.7519delC(p.Pro2508Leufs*35)	Compound heterozygous	U/LP	0/1	Moderate	Down-sloping
M2018	F	Han	8	0	c.1179insC(p.Glu396Argfs*36)	c.10419_10423delCAGCT(p.Ser3474Profs*42)	Compound heterozygous	P/P	1/1	Profound	Undefined
M2027	F	Han	5	0	c.4441 T > C(p.Ser1481Pro)	c.4642G > A(p.Ala1548Thr)	Compound heterozygous	U/U	0/0	Profound	Total deafness
Y2082	F	Han	3	1	c.3700C > T(p.Gln1234Ter)	c.5036G > A(p.Cys1679Tyr)	Compound heterozygous	P/U	0/0	Profound	Total deafness
Y2084	M	Han	6	5	c.4676 T > C(p.Leu1559Ser)	c.9690 + 1G > A(splicing)	Compound heterozygous	U/P	0/1	Profound	Down-sloping
Y2103	F	Han	2	0	c.4987G > A(p.Asp1663Asn)	c.9358C > T(p.Gln3120Ter)	Compound heterozygous	U/P	0/1	Severe	Flat
Y2107	F	Han	6	0	c.8324G > T(p.Arg2775Leu)	c.9941del(p.Tyr3314Serfs*9)	Compound heterozygous	P/P	0/1	Profound	Total deafness
Y2109	F	Han	4	0	c.2231C > A(p.Ser744Ter)	c.9690 + 1G > A(splicing)	Compound heterozygous	P/P	1/1	Profound	Total deafness
Y2110	M	Han	8	0	c.3524dupA(p.Ser1176Valfs*13)	c.6611G > A(p.Arg2204His)	Compound heterozygous	P/LP	1/0	Profound	Total deafness
M2112	M	Han	4	0	c.3829C > T(p.Gln1277Ter)	c.5134-1G > A(splicing)	Compound heterozygous	P/P	1/1	Profound	Total deafness
M2177	F	Manchu	8	0	c.8151delC(p.Leu2718Cysfs*20)	c.10291_10305delGCCCCTTGCATCCTT(p.3431_3435delAPCIL)	Compound heterozygous	LP/U	1/1	Profound	Total deafness
M2194	M	Han	21	0	c.5360G > A(p.Arg1787Lys)	c.6510-1G > T(splicing)	Compound heterozygous	LP/P	0/1	Profound	Total deafness
M2218	M	Han	4	3	c.3524dupA(p.Ser1176Valfs*13)	c.10250_10252del(p.Ser3417del)	Compound heterozygous	P/LP	1/1	Moderate	Undefined
Y2123	M	Han	3	0	c.4596 + 1G > A(splicing)	c.4793A > G(p.Asn1598Ser)	Compound heterozygous	P/U	1/0	Profound	Total deafness
Y2128	M	Han	2	0	c.220_221del(p.Arg74Glufs*153)	c.9478C > T(p.Leu3160Phe)	Compound heterozygous	P/U	1/0	Severe	Flat
Y2129	F	Han	31	1	c.4310A > G(p.Tyr1437Cys)	c.8324G > A(p.Arg2775His)	Compound heterozygous	LP/U	0/0	Profound	Undefined
Y2138	M	Han	2	0	c.3136delC(p.Lys1048Argfs*10)	c.4441 T > C(p.Ser1481Pro)	Compound heterozygous	LP/U	1/0	Severe	Flat

^a^M: Male, F: Female

^b^P pathogenic, LP likely pathogenic, U unknown significance

^c^1 truncating variant, 0 non-truncating variant

**Table 3:** The severity of HL with different pathogenicity of variants

Pathogenicity of variant*	Severity of HL					
	Mild	Moderate	Moderately severe	Severe	Profound	Total
P/P				3	14	17
P/LP		1	1	1	11	14
P/U	1		1	4	17	23
LP/LP			1		3	4
LP/U		2		1	14	17
U/U			1	3	5	9
Total	1	3	4	12	64	84

P: Pathogenic; LP: Likely pathogenic; U: Uncertain significance

Variants with HIGH impact (e.g., frameshift variants, splice variants, stop gain variants, etc.) were counted as protein-truncating variants (PTVs) [19]. The 81 cases were divided into groups according to the number of PTVs: 2 truncating (34 cases); 1 truncating and 1 non-truncating (37 cases); 2 non-truncating variants (13 cases) (Table 4). We compared the severity of HL among the groups. The 2 non-truncating variants group had the least number of cases (12/81), and most of them (10/12) were with profound NSHL. Thus, we suggested that cases with the monoallelic or biallelic truncating *MYO15A* variant may increase the risk of HL.

**Table 4:** The severity of HL cases with different numbers of truncating variants

Number of truncating variant*	Severity of HL					
	Mild	Moderate	Moderately severe	Severe	Profound	Total
1/1		1	2	3	27	33
1/0	1	2		9	27	39
0/0			2		10	12
Total	1	3	4	12	64	84

^*^1 Truncating variant; 0 Non-truncating variant

**Fig. 3:**
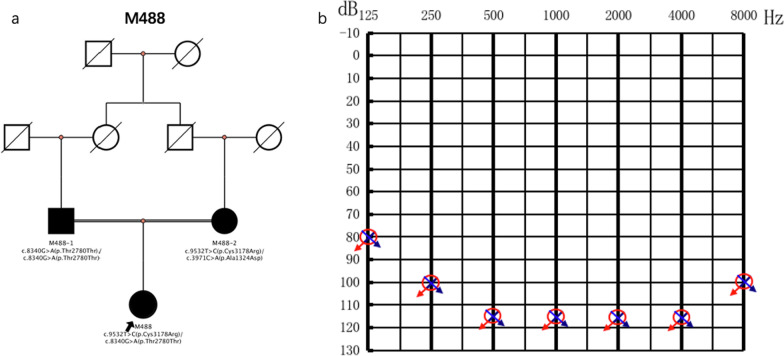
The audiograms and the pedigree of case M488. **a** Pedigree of case M488 and her family members. **b** All case M488 and her parents had the same profound HL of total deafness type

Although synonymous variation is generally considered as non-pathogenic, the variant c.8340G > A(p.Thr2780Thr) identified in the case M488 (Fig. 3) was considered to be pathogenic (PVS1_Very Strong, PM2_Moderate, PP5_Supporting) based on the ACMG/AMP classification in our cohort. In the NCBI ClinVar database, it was shown that the c.8340G > A (p.Thr2780Thr) predicted loss of exon 45 and led to a stop codon. (National Center for Biotechnology Information. ClinVar; [VCV000236038.1], https://www.ncbi.nlm.nih.gov/clinvar/variation/VCV000236038.1 (accessed Sept. 20, 2021).)

### Clinical findings

Among the 2263 cases from 1842 families with NSHL included in this study, including 1215 males and 1048 females. Age ranged from a few days after birth to 65 years with a mean age of 15.01 ± 13.67 years and the median age of 7.92 years. In our cohort, 1654 cases had prelingual HL and 609 had postlingual HL; 71 cases were mild, 238 were moderate, 179 were moderately severe, 512 were severe and 1263 were profound HL.

There were 81 (3.58%, 81/2263) cases from 74 families identified with at least one *MYO15A* pathogenic or likely pathogenic variant, or uncertain significant variant. Among them, 45 were males and 36 females, aged from 3 months to 43 years, with an average age of 10.41 ± 10.32 years. The ethnic distribution among the cases was as follows: one case was belonged to Korean ethnic group, one of Manchu, one of Tujia, while the others were all Han. None of the participants had a history of using aminoglycoside antibiotics.

**Fig. 4:**
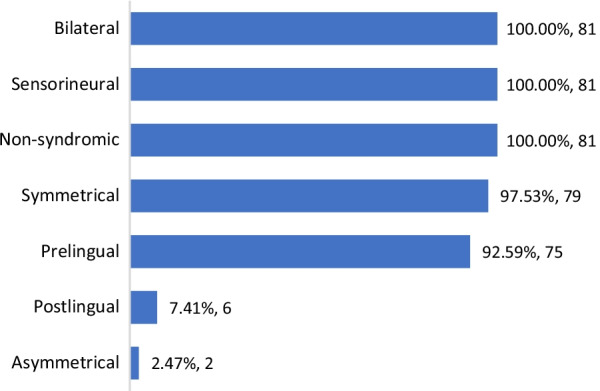
Audiological phenotype of *MYO15A*-related HL

Most of the audiological assessments and clinical history of the affected members showed a prelingual (92.59%, 75/81), symmetrical (97.53%, 79/81), bilateral (100%, 81/81), non-syndromic (100%, 81/81), sensorineural (100%, 81/81) HL (Fig. 4). Only a few showed a postlingual (7.41%, 6/81) and asymmetrical (2.47%, 2/81) HL. Analysis of the high-resolution CT scan of the temporal bone in the affected members showed a normal middle and inner ear structure.

The cases showed large variations in the degree of HL. The degree of HL was profound in 61 cases (75.30%, 61/81), severe in 12 (14.81%, 12/81), moderately severe in 4 (4.94%, 4/81), moderate in 3 (3.70%, 3/81) and mild in 1 (1.22%, 1/81). The last had the right ear with a mild HL and the left ear with a profound HL. Audiogram forms showed 6 cases with a flat type, 50 cases with total deafness, 10 cases with a descending type, whereas 24 remained undefined.

**Fig. 5:**
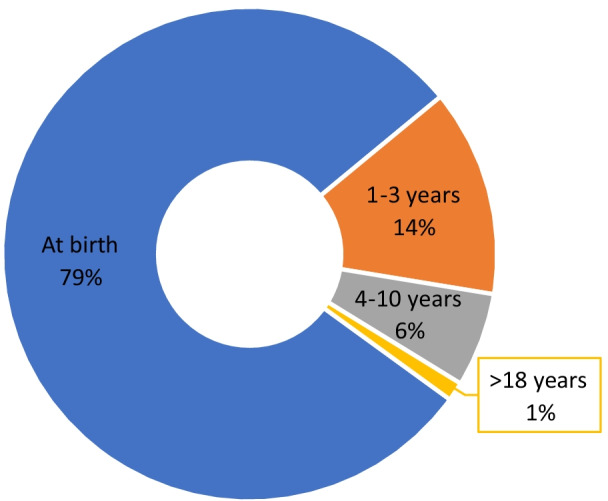
Age of onset of *MYO15A*-related HL

The age of onset among cases ranged from a few days after birth to 41 years. The hearing loss in 79.01% (64/81) of the cases appeared at birth, in 13.58% (11/81) was detected during the first 1–3 years, in 6.17% (5/81) HL arose around the age of 4–10 years, in 1.23% (1/81) was reported after 18 years (with severe deafness in the left ear and moderate deafness in the right ear, especially at the age of 41). (Fig. 5).

**Fig. 6:**
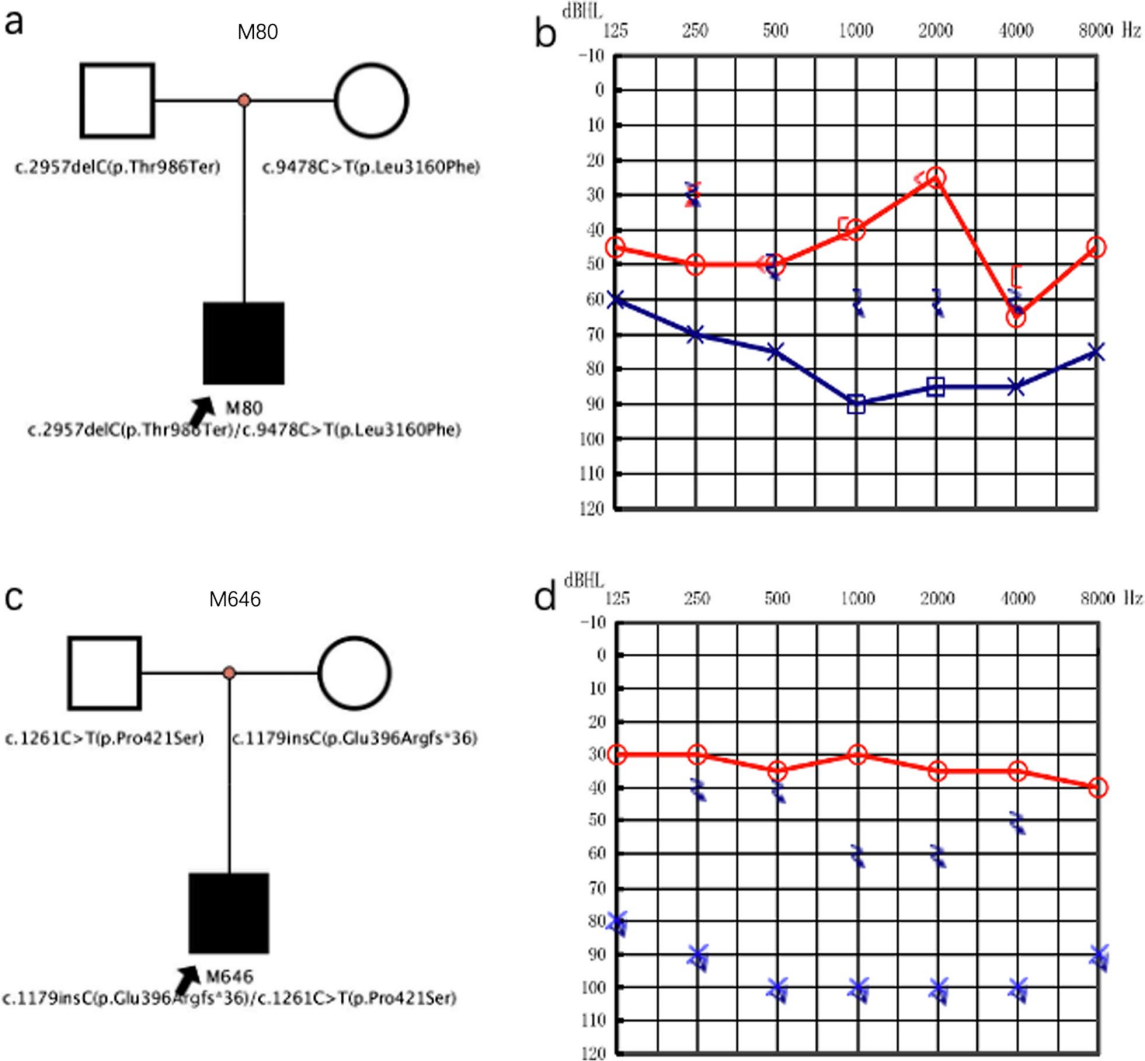
The audiograms and the pedigree of case M80 (**a**, **b**) and M646 (**c**, **d**)

In our study, it was found that the genotype–phenotype correlation between the variants in the *MYO15A* gene and the HL in some cases was different from that of the others. For example, the cases M80 and M646 with an asymmetric unilateral severe deafness bore the compound heterozygous variants c.2957delC(p.Thr986fs)/c.9478C < T(p.Leu3160Phe) and c.1179insC(p.Glu396Argfs*36)/c.1261C > T (p.Pro421Ser), respectively. (Fig. 6).

Affected subjects also showed progression with the different onset of HL. Case M623 with c.10251_10253delCTT homozygous variants was found in this study, who passed the hearing screening at birth, but was diagnosed with HL at the age of 3 and the HL demonstrated a progressive trait. His brother carrying the same c.10251_10253delCTT homozygous variant showed severe bilateral sensorineural HL at the age of 3 years.

Through the telephone follow-up of 56 *MYO15A*-related cases, the effect of using hearing aids and cochlea implants was satisfactory in most of the participants.

## Discussion

Mutations in *MYO15A* were initially identified in HL individuals of consanguineous families from Bengkala, Bali in 1995 [12, 13]. Screening for the reported variants in the *MYO15A* gene with 66 exons was a very difficult and expensive task at that time. Therefore, the *MYO15A* gene was rarely sequenced in familial segregated deafness unless significant genetic linkage data implicated the presence of the DFNB3 locus. Instead, efforts were invested in screening for variations in smaller genes that have been identified as important contributors to inherited HL in humans, such as *GJB2*, which has only one protein-coding exon. The widespread contribution of *MYO15A* mutations on human HL was not recognized until the NGS became cost-effective and widely adopted around the world [20]. Now, mutations in *MYO15A* are a widely recognized cause of recessively inherited NSHL globally. More than 200 *MYO15A* variants have previously been reported ranging along with the domains and motifs of the encoded by *MYO15A* protein myosin XVA (Table 5) [8, 11, 13, 20–68].

**Table 5:** Overview of published variants of the *MYO15A* in NSHL patients

Exon	Domain	Nucleotide Change	Amino Acid Change	Age of Onset	Hearing Level^a^	ACMG Classification^b^	Origin of Family	Reference
2	N-terminal	c.373_374delCG	p.Arg125Valfs*101	–	Profound	–	Ashkenazi, Jewish	Brownstein (2011)
2	N-terminal	c.419del	p.Lys140Serfs*304	–	Profound	–	–	Zhang (2019)
2	N-terminal	c.453_455delCGAinsTGGACGCCTGGTCGGGCAGTGG	p.Glu152Glyfs*81	Progressive	Mild and Profound	–	Qatar	Vozzi (2014)
2	N-terminal	c.514C > T	p.Leu172Phe	–	–	LP	Japan	Miyagawa (2013)
2	N-terminal	c.535G > T	p.Glu179Ter	Congenital	Moderate and severe	P	Korea, Japan	Park (2014), Miyagawa (2015)
2	N-terminal	c.554G > A	p.Gly185Asp	–	–	U	Japan	Miyagawa (2013)
2	N-terminal	c.613 T > C	p.Phe205Leu	–	–	U	Japan	Miyagawa (2013)
2	N-terminal	c.625G > T	p.Glu209Ter	–	Severe to profound	P	–	Zhang (2019)
2	N-terminal	c.671A > G	p.Tyr224Cys	–	–	U	Japan	Miyagawa (2013)
2	N-terminal	c.742C > G	p.Arg248Gly	–	–	P	–	Rehman (2016)
2	N-terminal	c.855dup	p.Pro286Serfs*15	Congenital	Severe to profound	P	China	Zhang (2019)
2	N-terminal	c.867C > G	p.Tyr289Ter	Congenital or prelingual, progressive	Moderate to severe/R	P	Turkey	Cengiz (2010)
2	N-terminal	c.1047C > A	p.Tyr349Ter	–	–	P	Russian	Imtiaz (2011)
2	N-terminal	c.1047C > T	p.Tyr349 =	–	–	LB	Saudi Arabia	Sloan-Heggen (2015), Imtiaz (2011)
2	N-terminal	c.1137delC	p.Tyr380Metfs*65	Prelingual progressive	Normal between 0.125 and 0.25 kHz/S	P	German	Vona (2014)
2	N-terminal	c.1171_1177dupGCCATCT	p.Tyr393Cysfs*41	Congenital	Severe to profound	P	Oman	Palombo (2017)
2	N-terminal	c.1185dupC	p.Glu396Argfs*36	10–14 y congenital	Moderate to profound/R	P	Pakistan, Japan	Bashir (2012), Miyagawa (2013)
2	N-terminal	c.1223C > T	p.Ala408Val	–	–	P	–	Brownstein (2014)
2	N-terminal	c.1387A > G	p.Met463Val	Congenital	Severe to profound/R	C	Iran	Fattahi (2012)
2	N-terminal	c.1454 T > C	p.Val485Ala	–	–	C	–	Sloan-Heggen (2015)
2	N-terminal	c.1634C > T	p.Ala545Val	–	–	C	–	Sloan-Heggen (2015)
2	N-terminal	c.1651G > A	p.Ala551Thr	Congenital	Severe to profound	U	-	Zhang (2019)
2	N-terminal	c.2456C > A	p.Ser819Ter	Congenital	Severe to profound	LP	Pakistan	Richard (2019)
2	N-terminal	c.2516del	p.Pro839Argfs*24	–	–	P	Iran	Sloan-Heggen (2015)
2	N-terminal	c.2759G > A	p.Trp920Ter	Congenital	Moderate	–	Iran	Sloan-Heggen (2015)
2	N-terminal	c.3020C > A	p.Pro1009His	Congenital	–	–	China	Yang (2013)
2	N-terminal	c.3026C > A	p.Pro1009His	Congenital	–	C	China	Yang (2013)
2	N-terminal	c.3313G > T	p.Glu1105Ter	Congenital	Profound	P	Pakistan	Nal (2007), Miyagawa (2013)
2	N-terminal	c.3334delG	p.Arg1112fs*1124	Congenital	Mild to Profound/R	–	Pakistan	Nal (2007), Miyagawa (2013)
2	N-terminal	c.3505C > T	p.Arg1169Ter	Congenital	Severe to profound	P	Pakistan	Richard (2019)
2	N-terminal	c.3524dupA	p.Ser1175Valfs*1188	Congenital	Severe/R	P	China	Li (2016)
2	N-terminal	c.3524dup	p.Ser1176Valfs*14	Congenital	Mild	P	China	Zhang (2019)
2	Motor	c.3685C > T	p.Gln1229Ter	Congenital	Profound	P	Pakistan	Liburd (2001)
Intron 4	Motor	c.3756 + 1G > T	p.Asp1232fs*1241	Congenital	Profound	P	Pakistan	Liburd (2001)
4	Motor	c.3742C > T	p.Arg1248Thr	Congenital	Severe	U	China	Zhang (2019)
4	Motor	c.3758C > T	p.Thr1253Ile	Congenital	Severe to profound	P	India	Nal (2007)
Intron 5	Motor	c.3866 + 1G > A	p.Thr1253fs*1277	Congenital	Moderate to profound	P	Pakistan	Nal (2007), Naz (2017)
5	Motor	c.3844C > T	p.Arg1282Trp	Congenital	Severe to profound	U	Netherlands	Neveling (2013)
6	Motor	c.3866dupC	p.His1290Alafs*25	Congenital	Severe to profound	U	China	Bai (2019)
6	Motor	c.3871C > T	p.Leu1291Phe	Congenital	Severe	P	–	Zhang (2019)
6	Motor	c.3892G > A	p.Ala1298Thr	Congenital	Mild to Severe/R	–	China	Gu (2015)
6	Motor	c.3932 T > C	p.Ile1311Thr	–	–	LP	–	Zhang (2019)
6	Motor	c.3944G > A	p.Gly1315Glu	–	–	P	–	Zhang (2019)
8	Motor	c.4072G > A	p.Gly1358Ser	Second decade	Moderate and severe		Japan	Miyagawa (2015)
9	Motor	c.4176C > A	p.Tyr1392Ter	–	Severe to profound	P	Pakistan, Iran	Nal (2007), Sloan-Heggen (2015)
9	Motor	c.4198G > A	p.Val1400Met	Congenital or prelingual	Severe to profound	P and L	Turkey	Manzoli (2016), Cengiz (2010)
11	Motor	c.4216G > A	p.Glu1406Lys	–	–	LP	Japan	Miyagawa (2013)
10	Motor	c.4240G > A	p.Glu1414Lys	–	–	P	Palestinian, Arab	Brownstein (2011)
11	Motor	c.4252G > A	p.Gly1418Arg	Congenital	Moderate	P	China	Zhang (2019)
10	Motor	c.4273C > T	p.Gln1425Ter	–	–	P and LP	Turkey	Miyagawa (2015)
11	Motor	c.4310A > G	p.Tyr1437Cys	Postlingual childhood	Mild moderate	U	Iran	Sloan-Heggen (2015)
11	Motor	c.4313 T > C	p.Leu1438Pro	Congenital	Severe to profound	P	–	Zhang (2019)
Intron 11	Motor	c.4320 + 1G > A	–	–	–	P and LP	Korea	Park (2014), Woo (2013)
12	Motor	c.4322G > T	p.Gly1441Val	Congenital	Mild and Severe/R	P and LP	Japan; China	Miyagawa (2013), Gu (2015), Moteki (2016)
11	Motor	c.4351G > A	p.Asp1451Asn	–	Severe to profound	P and LP	India	Nal (2007)
11	Motor	c.4441 T > C	p.Ser1481Pro	Congenital or prelingual	Severe to profound	P and LP	Turkey	Cengiz (2010), Diaz-Horta (2012)
13	Motor	c.4519C > T	p.Arg1507Ter	Congenital	Severe to profound	P	Iran	Sarmadi (2020)
13	Motor	c.4528C > T	p.Gln1510Ter	–	–	P and LP	Pakistan	Sloan-Heggen (2015)
13	Motor	c.4642G > A	p.Ala1548Thr	Congenital	Severe to profound	P	China	Chen (2016)
13	Motor	c.4652C > A	p.Ala1551Asp	–	–	–	Turkey	Miyagawa (2015)
Intron 14	Motor	c.4655 + 1G > A	–	–	–	P and LP	Iran	Sloan-Heggen (2015)
15	Motor	c.4666G > A	p.Ala1556Thr	–	mild	U	China	Zhang (2019)
15	Motor	c.4669A > G	p.Lys1557Glu	–	Severe to profound	–	Pakistan	Nal (2007)
15	Motor	c.4747 T > C	p.Ser1583Pro	Congenital	Profound	–	China	Zhang (2019)
15	Motor	c.4777G > A	p.Glu1593Lys	–	–	U	–	Sloan-Heggen (2015)
15	Motor	c.4780G > C	p.Asp1594His	Congenital	Severe to profound	P	–	Zhang (2019)
15	Motor	c.4823C > A	p.Ala1608Glu	Congenital	Profound	–	China	Zhang (2019)
16	Motor	c.4828G > A	p.Glu1610Lys	–	–	U	Japan	Miyagawa (2013)
17	Motor	c.4888C > G	p.Arg1630Gly	–	–	U	Japan	Miyagawa (2013)
17	Motor	c.4898 T > C	p.Ile1633Thr	Congenital	Severe/R	U	China, Pakistan	Gu (2015), Rehman (2016)
17	Motor	c.4904_4907delGAG	p.Gly1637del	Postlingual	Severe to profound	P and LP	Iran	Fattahi (2012)
17	Motor	c.4952C > T	p.Ser1651Leu	–	–	U	–	Sloan-Heggen (2015)
16	Motor	c.4998G > A	p.Cys1666Ter	–	–	–	Tunisia	Belguith (2009)
18	Motor	c.5087dup	p.Pro1697Alafs*2	Congenital	Severe to profound	P	–	Zhang (2019)
18	Motor	c.5117_5118GC > TT	p.Leu1706Val	–	Severe to profound	–	Pakistan	Belguith (2009)
19	Motor	c.5141A > T	p.Leu1714Met	Congenital	Moderate	U	–	Zhang (2019)
18	Motor	c.5189 T > C	p.Gly1730Pro	–	Severe to profound	–	Pakistan	Nal (2007)
19	Motor	c.5203C > T	p.Arg1735Trp	–	–	U	–	Zhang (2019)
19	Motor	c.5212-2A > G	–	–	–	U	Turkey	Atik (2015)
20	Motor	c.5287C > T	p.Arg1763Trp	–	–	B	Netherlands	Neveling (2013)
20	Motor	c.5305A > G	p.Thr1769Ala	Congenital	Severe to profound/R	–	Iran	Fattahi (2012)
20	Motor	c.5336 T > C	p.Leu1779Pro	Congenital	Profound	U	Algerian	Ammar-Khodja (2015)
22	Motor	c.5417 T > C	p.Leu1806Pro	–	–	P	–	Zhang (2019)
22	Motor	c.5421delT	p.Phe1807Leufs*6	Congenital	Severe to profound /R	–	Iran	Fattahi (2012)
21	Motor	c.5492G > T	p.Gly1831Val	–	Severe to profound	P	Turkey	Kalay (2007)
22	Motor	c.5504G > A	p.Arg1835His	Postlingual, progressive	Mild to severe/R	–	Korea	Chang (2018)
22	Motor	c.5507 T > C	p.Leu1836Pro	Congenital	Profound	–	China	Zhang (2019)
Intron 22	Motor	c.5650-1G > A	p.Ala1884Ter	–	–	–	Turkey	Duman (2011)
24	Motor	c.5692C > T	p.Arg1898Ter	–	–	U	China	Zhang (2019)
23	Motor	c.5808_5814delCCGTGGC	p.Arg1937Thrfs*10	Congenital or prelingual	Severe to profound	P and LP	Turkey	Cengiz (2010)
23	IQ3	c.5809C > T	p.Arg1937Cys	–	–	U	Iran, Pakistan	Rehman (2016), Sloan-Heggen (2015)
23	IQ3	c.5810G > A	p.Arg1937His	Postlingual or congenital	Mild and severe to profound/R	P and LP	Iran	Fattahi (2012), Sloan-Heggen (2015)
23	IQ3	c.5835 T > G	p.Tyr1945Ter	Congenital	Profound	P	Korea	Chang (2015)
25	IQ Motif	c.5925G > A	p.Trp1975Ter	Congenital	Severe to profound/R	C	Iran	Fattahi (2012)
Intron 26	IQ Motif	c.5964 + 3G > A	–	–	–	U	China	Gao (2013)
27	IQ Motif	c.5977C > T	p.Arg1993Trp	–	–	U	China	Zhang (2019)
27	IQ Motif	c.5978G > A	p.Arg1993Gln	First decade/Postlingual	Mild and severe/R	C	Japan	Miyagawa (2015)
28	IQ Motif	c.6052G > A	p.Gly2018Arg	–	Mild	B	–	Zhang (2019)
27	-	c.6061C > T	p.Gln2021Ter	–	Severe to profound	–	Pakistan	Nal (2007)
27	IQ Motif	c.6146C > A	p.Pro2049His	Congenital	Severe to profound	P	–	Zhang (2019)
Intron 27	IQ Motif	c.6178-2A > G	–	Congenital	Severe to profound	P	Pakistan	Rehman (2016)
28	MyTH4	c.6217C > T	p.Pro2073Ser	Congenital	Profound	U	Iran	Shearer (2009)
29	MyTH4	c.6306_6307insG	p.Ala2104Cysfs*18	–	–	–	China	Yang (2013)
29	MyTH4	c.6331A > T	p.Asn2111Tyr	Congenital	Profound	P	Iran	Wang (1998)
29	MyTH4	c.6337A > T	p.Ile2113Phe	Congenital	Profound	P	Indonesia	Wang (1998)
29	MyTH4	c.6340G > A	p.Val2114Met	–	–	P	China	Yang (2013)
30	MyTH4	c.6371G > A	p.Arg2124Gln	Congenital	Mild and severe to profound/R	L	Iran	Shearer (2009)
30	MyTH4	c.6437G > A	p.Arg2146Gln	Postlingual	Mild and severe	P and LP	Korea; Iran	Sloan-Heggen (2015), Woo (20,130
30	MyTH4	c.6436C > T	p.Arg2146Trp	–	Mild	U	–	Zhang (2019)
30	MyTH4	c.6487delG	p.Ala2153Profs*100	Prelingual	Mild to profound/R	P and LP	Japan	Miyagawa (2015)
30	MyTH4	c.6589C > T	p.Gln2197Ter	–	–	P	Pakistan	Rehman (2016)
30	MyTH4	c.6614C > T	p.Thr2205Ile	Congenital	Moderate	U	North America	Liburd (2001)
31	MyTH4	c.6634G > A	p.Glu2212Leu		Moderate	U	–	Zhang (2019)
32	-	c.6703 T > C	p.Ser2235Pro	Second decade/postlingual	Moderate/R	U	Japan	Miyagawa (2015)
31	-	c.6731G > A	p.Gly2244Glu	Prelingual	Severe to profound	P and LP	Pakistan, Japan	Nal (2007), Miyagawa (2015)
Intron 32	-	c.6764 + 2 T > A	–	–	–	P and LP	Netherlands	Sloan-Heggen (2015), Neveling (2013)
33	-	c.6787G > A	p.Gly2263Ser	–	–	U	–	Sloan-Heggen (2015)
31	-	c.6796G > A	p.Val2266Met	–	Severe to profound	U	Pakistan, Turkey	Nal (2007)
33	-	c.6845A > G	p.Tyr2282Cys	–	–	U	–	Zhang (2019)
33	-	c.6893G > A	p.Arg2298Gln	–	–	LP	–	Sloan-Heggen (2015)
Intron 33	-	c.6956 + 9C > G	–	–	–	U	–	Yang (2013)
34	-	c.7047del	p.Tyr2350Thrfs*67	Congenital	Profound	P	–	Zhang (2019)
35	-	c.7124_7127delACAG	p.Asp2375Valfs*29	Prelingual progressive	Severe	P and LP	Germany	Vona (2014)
Intron 36	-	c.7395 + 3G > C	–	–	Severe to profound	U	Tunisia	Belguith (2009), Riahi (2014)
35	-	c.7207G > T	p.Asp2403Tyr	Congenital	Profound	P	Palestinian Territories	Shahin (2010)
36	-	c.7226del	p.Pro2409Glnfs*8	–	–	P	Puerto Rico	Sloan-Heggen (2015), Bademci (2016)
39	-	c.7550C > G	p.Thr2517Ser	Congenital	Mild moderate asymmetric	U	Iran	Sloan-Heggen (2015)
39	-	c.7636C > T	p.Gln2546Ter	Congenital	Profound	U	–	Zhang (2019)
40	-	c.7679G > A	p.Arg2560Gln	–	–	U	–	Sloan-Heggen (2015)
40	-	c.7708_7709insCA	p.Gln2571Hisfs*35	Congenital	Profound	–	China	Zhang (2019)
39	SnAPC2 like	c.7801A > T	p.Lys2601Ter	Congenital	Profound	P	India	Wang (1998)
41	-	c.7822G > A	p.Asp2608Asn	Congenital	Profound	U	China	Zhang (2019)
42	-	c.7894G > T	p.Val2632Leu	–	–	U	–	Bademci (2016)
41	SnAPC2 like	c.7982C > A	p.Ser2661Ter	–	–	–	Turkey	Duman (2011)
43	-	c.7990C > A	p.Pro2664Thr	–	–	LB	–	Zhang (2019)
43	-	c.8033_8056del	p.Asn2678Ter	Congenital	Severe	–	China	Zhang (2019)
43		c.8050 T > C	p.Tyr2684His	Congenital	Severe	U	–	Zhang (2019)
44	FERM	c.8077del	p.Leu2693Cysfs*45	Congenital	Mild to profound	–	China	Zhang (2019)
44	FERM	c.8090 T > C	p.Val2697Ala	Congenital	Severe	P	–	Zhang (2019)
46	FERM	c.8148G > T	p.Gln2716His	Congenital	Profound	P	Pakistan	Liburd (2001)
43	FERM	c.8158G > C	p.Asp2720His	–	Moderate to profound	P and LP	Pakistan	Nal (2007), Naz (2017)
43	-	c.8183G > A	p.Arg2728His	Congenital	–	P and LP	Jewish, China	Yang (2013), Brownstein (2011)
43	-	c.8198A > C	p.Glu2733Ala	Congenital	Profound	–	Japan	Miyagawa (2015)
45	-	c.8222 T > C	p.Phe2741Ser	–	–	P	–	Zhang (2019)
Intron 45	-	c.8224 + 3A > G	splice site	–	–	LP	Pakistani	Richard (2019)
46	-	c.8309_8311del	p.Glu2770del	–	–	P and LP	Turkey, Iran	Sloan-Heggen (2015), Bademci (2016)
43	-	c.8324G > A	p.Arg2775His	–	–	–	China	Yang (2013)
46	-	c.8340G > A	p. Thr2780Thr	Congenital	Profound	P	Israel	Danial-Farran (2018)
47	-	c.8375 T > C	p.Val2792Ala	–	–	P	China	Gao (2013)
47	FERM	c.8445_8448delCCTG	p.Val2815Valfs*10	Congenital	Severe to profound	P	Iran	Sarmadi (2020)
47	FERM	c.8450G > A	p.Arg2817His	Congenital	Mild to severe/R	U	China	Gu (2015)
47	FERM	c.8457C > G	p.Tyr2819Ter	–	–	P	–	Zhang (2019)
48	FERM	c.8467G > A	p.Asp2823Asn	Congenital	Moderate to profound/R	P and LP	Iran	Fattahi (2012), Sloan-Heggen (2015)
49	SH3	c.8707C > T	p.Arg2903Ter	Congenital	Profound	U	–	Zhang (2019)
50	SH3	c.8725G > A	p.Gly2909Ser	Congenital	Profound	P	–	Zhang (2019)
48	SH3	c.8767C > T	p.Arg2923Ter	–	–	P and LP	China	Woo (2013)
50	SH3	c.8771G > A	p.Arg2924His	–	Mild and severe	LB	–	Zhang (2019)
50	SH3	c.8791del	p.Trp2931Glyfs*103	Congenital	Profound		China	Zhang (2019)
51	SH3	c.8812G > A	p.Gly2938Arg	Congenital	Mild moderate asymmetric	U	Iran	Sloan-Heggen (2015)
49	SH3	c.8821_8822insTG	p.Val2940fs*3034	Congenital	Severe to profound	–	Pakistan	Nal (2007)
49	SH3	c.8899dup	p.Arg2967ProfsTer33	Congenital	Profound	–	Germany	Budde (2020)
49	SH3	c.8899C > T	p.Arg2967Ter	Congenital	Profound	–	Germany	Budde (2020)
Intron49	-	c.8968-1G > C	–	–	Profound	P	Turkey	Kalay (2007)
52	-	c.9083 + 6 T > A	–	Congenital	Profound	P	Israel	Danial-Farran (2018)
Intron53	-	c.9229 + 1G > A	–	–	Severe to profound	–	Tunisia	Belguith (2009)
54	MyTH4	c.9221 T > C	p.Met3074Thr	–	–	U	–	Zhang (2019)
56	MyTH4	c.9316dupC	p.H3106Pfs*2	Congenital	Severe to profound	P	China	Xia (2015)
57	MyTH4	c.9400C > T	p.Arg3134Ter	–	–	P	–	Zhang (2019)
57	MyTH4	c.9408G > C	p.Trp3136Cys	–	–	U	–	Zhang (2019)
57	MyTH4	c.9413 T > A	p.Leu3138Gln	Congenital or prelingual	Moderate to Profound/ R	P and LP	Japan	Miyagawa (2015)
59	MyTH4	c.9478C > T	p.Leu3160Phe	Congenital	Severe to profound/ R	U	Pakistan; Japan	Nal (2007), Miyagawa (2013), Miyagawa (2015)
57	MyTH4	c.9517G > A	p.Gly3173Arg	First decade/postlingual	Mild to severe/R	–	Japan	Miyagawa (2015)
58	MyTH4	c.9534C > G	p.Cys3178Trp	Congenital	Severe to profound	P	–	Zhang (2019)
58	MyTH4	c.9571C > T	p.Arg3191Cys	Congenital	Severe to profound	P	China	Zhou (2019)
58	MyTH4	c.9572G > A	p.Arg3191His	Congenital	Severe to profound	P	–	Zhang (2019)
57	MyTH4	c.9584C > G	p.Pro3195Arg	prelingual	Moderate to severe	–	Iran	Mehregan (2019)
Intron 58	MyTH4	c.9611_9612 + 8del TGGTGAGCAT	p.Leu3204Cysfs*17	Congenital	–	P	Iran	Akbariazar (2019)
59	MyTH4	c.9620G > A	p.Arg3207His	–	–	U	–	Bademci (2016)
60	FERM	c.9781A > T	p.Asn3261Tyr	–	–	U	–	Miyagawa (2013)
60	FERM	c.9790C > T	p.Gln3264Ter	Postlingual, progressive	Mild to severe/R	–	Korea	Chang (2018)
61	FERM	c.9908A > G	p.Lys3303Arg	–	–	U	–	Sloan-Heggen (2015)
65	FERM	c.9958_9961delGACT	p.Asp3320Thrfs*2	First decade	Severe to profound	P	Brazil	Lezirovitz (2008)
65	FERM	c.9995_10002dupGCCGGCCC	p.Ser3335Alafs*121	Congenital or prelingual	Severe to profound	P and LP	Turkey	Cengiz (2010)
63	FERM	c.10181C > T	p.Ala3394Val	Congenital	Severe to profound	U	–	Zhang (2019)
63	FERM	c.10202G > A	p.Arg3401His	Postlingual childhood	Mild moderate	P	Iran	Sloan-Heggen (2015)
64	FERM	c.10245_10247delCTC	p.Ser3417del	Postlingual, progressive	Severe/R	P	Korea	Chang (2018), Miyagawa (2015)
64	FERM	c.10249_10251delTCC	p.Phe3417del	Congenital	Profound	P	Japan	Miyagawa (2015)
64	FERM	c.10258_10260del	p.Phe3420del	Congenital	Profound	P	China	Zhang (2019)
64	FERM	c.10263C > G	p.Ile3421Met	10–19 y/ Postlingual, progressive	Moderate to severe/R	U	Japan/Korea	Chang (2018), Miyagawa (2015)
65	FERM	c.10394G > A	p.Arg3465Gln	–	–	U	–	Sloan-Heggen (2015)
66	FERM	c.10474C > T	p.Gln3492Ter	–	Severe to profound	P	Pakistan	Nal (2007)
66	FERM	c.10572dup	p.Ser3525fs*79	–	–	P	–	Zhang (2019)
66	FERM	c.10573delA	p.Ser3525fs*29	Prelingual	Severe to profound	P	Brazil	Lezirovitz (2008)

^a^R residual hearing of low frequencies, S steeply sloping to severe hearing loss

^b^P pathogenic, LP likely pathogenic, LB likely benign, B benign, U unknown significance

^c^Conflicting interpretations of pathogenicity

Many studies analyzed the mutations in the *GJB2* and *SLC26A4* genes among cases with NSHL in different parts of the world. The obtained results demonstrated that the prevalence of the variants in *GJB2* and *SLC26A4* in HL accounted for about 15% to 25% and 2% to 12.6%, respectively, all dependent on the region localized [69]. The reported frequency of *MYO15A* variations in HL was 1.1% to 28% in respect to the different regions [70]. Besides Farjami et al. [70] reported that the *MYO15A* variant frequency in NSHL was 4.9% considering the variant rate of the *GJB2* gene of 20%. In our study, the estimated prevalence of *MYO15A* variants in NSHL was 3.58%, which was similar to Farjami’s report. Moreover, Farjami et al. [70] proposed a total of 192 recessive *MYO15A* variants related to HL. The evaluated proportions of the various types of variants detected by him were similar to those noticed in our study. The composition of the detected variant types was similar in the different intensities of the HL (see Fig. 1). The c.10245_ 10247delCTC variant was identified as the most recurrent HL variant in our cohort. According to the MAF of 0.000016 in the Exome Aggregation Consortium (ExAC) database, 0.000389 in East Asian population and 0.000281 in total population by gnomAD, the c.10245_10247delCTC had been previously reported pathogenic, causing ARNSHL in the Japanese, Korean and Chinese individuals [7, 21, 71]. Therefore, we suggest that this variant is the hotspot of the *MYO15A*-related NSHL variant in East-Asian populations.

In the past two decades, scholars worldwide have gradually made a progress in the understanding of the correlation between the genotype and the resultant phenotype of *MYO15A* variants. During the first decade, it was thought that the hearing phenotype of ARSNHL was congenital, bilateral, full-frequency, severe to profound sensorineural hearing loss (SNHL). In 2007, Nal et al. [22] reported for the first time that an N-terminal variant (p.Glu1112fs*1124) in the exon 2 of the *MYO15A* gene resulted in a mild hearing loss with residual hearing at low frequency. At that time, it was considered that the phenotype of the hearing loss in cases with *MYO15A* variants was closely related to the region where this gene variant was located. However, subsequent studies showed that the correlation between the genotype and phenotype of *MYO15A* seemed to be more complex. Notably, the congenital non-progressive NSHL was investigated as the main consequence of the *MYO15A* variants. Interestingly, in families with ARNSHL with the same *MYO15A* pathogenic variant, the degree of the hearing phenotype was different [23, 24]. Different hearing phenotypes of non-congenital binaural severe SNHL were reported. Except for the residual hearing in the low-frequency region [25], it also included congenital moderate and severe SNHL with descending hearing curve [22, 23, 26, 27], all-frequency moderate and severe SNHL [28], progressive high-frequency descending severe SNHL [29], delayed and progressive moderate and severe SNHL [7, 30]. Allelic heterogeneity is common in hearing loss and is associated with clinical phenotype heterogeneity [72]. The variability of phenotypes makes clinical diagnosis and variant interpretation in genetic hearing loss diagnosis and maintenance [17]. And in our study, we found that the *MYO15A* variants-related hearing phenotype of SNHL in China was similar to the previous reports.

Nevertheless, some reports showed that *MYO15A* pathogenic variants cause moderate-to-severe HL, although they previously had been presented to cause profound HL [7, 31]. We found three cases in our cohort with *MYO15A* variants in the N-terminal, motor and MyTH domains that were diagnosed with a subtle HL. The hypothesis indicated that the predicted amino acid substitutions of the intrinsically disordered N-terminal domain were structurally less menacing, leading to a subtler HL. Based on these results, we believe that *MYO15A* variants may be the cause leading to the postlingual onset of partial deafness, the molecular mechanism of which requires further investigation. The occurrence of this non-severe hearing phenotype may be related to the following factors: the weak pathogenicity of *MYO15A* alleles, the existence of modified genes to reduce the degree of HL, and the influence of environmental factors. In addition, the progress of technologies for genetic diagnosis recently has further enriched the phenotypic spectrum of *MYO15A*. In the past, linkage analysis was often used in the study of inbreeding hereditary ear families. Those cases with severe hearing phenotypes caused by homozygous variants were always given priority to be included in the relevant genetic research. However, with the use of the WES technology and Molecular Genetics techniques, sporadic and medium-sized families around the world started to be increasingly diagnosed, and more cases with compound heterozygous variants with different phenotypes were identified, which allowed the *MYO15A* variants to show more diverse phenotypic characteristics.

We have detected a synonymous variant in *MYO15A* which was considered as a pathogenic variant. Generally, synonymous variants are considered to be non-pathogenic and are not expected to change the function of proteins. In recent years, this paradigm has been challenged with the evidence that the changes in the codon usage affected the efficiency and speed of translation, which in turn modified the folding and function of proteins [73]. Furthermore, the possible pathogenic mechanism of the abnormal splice site caused by a single nucleotide substitution at the codon wobble site and its implication in the phenotypes of HL was often ignored. Its pathogenicity was suggested by both NCBI ClinVar and DVD databases. NCBI ClinVar database, c.8340G > A (p.Thr2780Thr) predicted loss of exon 45 (116 bp), leading to a stop codon 2803 of 3531, and was the only synonymous variant considered as pathogenic. The other synonymous variants were classified as benign, likely benign, uncertainly significant, and to some extent conflicting interpretations of pathogenicity (National Center for Biotechnology Information. ClinVar; [VCV000236038.1], https://www.ncbi.nlm.nih.gov/clinvar/variation/VCV000236038.1 (accessed Sept. 20, 2021).) Danial-Farran N et al. [32] reported that c.8340G > A (p.Thr2780Thr), in the last nucleotide of exon 46 eliminated the full exon inclusion isoform, indicating that this variant impaired splicing of exon 46. Therefore, c.8340G > A (p.Thr2780Thr) was also classified as PTV.

There was a limited understanding about the impact of *MYO15A* PTV across multiple phenotypes. In this study, the cases with biallelic non-truncating *MYO15A* variants commonly related with profound HL, and the cases with one or two truncating variants tended to show more prone to HL. Therefore, it suggested a correlation between genotype and phenotype in *MYO15A*-related NSHL.

Consistent with previous genetic studies, *MYO15A* variants are considered to play an important role in the pathogenesis of HL in China. There were several limitations of this study. First, the approach yet could not detect variants in the promoter or enhancer region and copy number variants. In addition, the follow-up time varies, some cases lack long-term follow-up results and objective evaluation, particularly the cochlear implant cases.

## Conclusion

In summary, we found that a total of 3.58% of the Chinese population with NSHL were related to *MYO15A* variants. *MYO15A* variants associated with NSHL were proven by NGS and validated by Sanger sequencing. Here, we report 78 novel and 24 reported *MYO15A* variants, which further enriched the *MYO15A* variant spectrum regarding the NSHL. Auditory features of the affected individuals were consistent with that previously reported for the recessive variants in the *MYO15A* gene. The hearing loss in most affected individuals was severe to profound, but in a few cases showed mild to moderate deafness. We suggest that the detected large variations in the phenotype of *MYO15A*-related NSHL might be correlated with the epigenetics and other factors that require further investigation. Noteworthy, screening for *MYO15A* variants in NSHL patients is of high necessity for efficient genetic diagnosis, patients’ counseling and clinical intervention.

## Supplementary Information

**Additional file 1 Table S1 L**ist of the 168 deafness genes and miRNA**.**

**Additional file 2. Table S2** Primer sets of Sanger sequencing used in this study.

**Additional file 3.** Detailed WES procedures.

## Data Availability

All data needed to evaluate the conclusions in the paper are present in the paper and/or the Supplementary Materials. Additional data related to this paper may be requested from the authors. The novel MYO15A variants were submitted to ClinVar under accession number: SUB10564921.

## Abbreviations

ABR: Auditory brainstem response
ACMG: Americal college of medical genetics and genomics
ARNSHL: Autosomal recessive non-syndromic sensorineural hearing loss
ASSR: Auditory steady state response
DPOAE: Distortion product otoacoustic emission
DVD: Deafness variation database
ExAC: Exome aggregation consortium
FERM: Fezrin, radixin, myosin
GATK: Genome analysis toolkit
HGMD: Human gene mutation database
HGVS: Human genome variation society
HL: Hearing loss
MAF: Minor allele frequency
MyTH4: Myosin-tail homology 4
NGS: Next-generation sequencing
NSHL: Non-syndromic sensorineural hearing loss
OAE: Otoacoustic emission
PCR: Polymerase chain reaction
PTA: Pure-tone audiometry
PTV: Protein-truncating variants
SNHL: Sensorineural hearing loss
VUS: Variant of uncertain significance
WES: Whole-exome sequencing
